# Chic. Society Physicians and Surgeons

**Published:** 1875-08

**Authors:** E. Warren Sawyer


### Reports of Societies.

### CHICAGO SOCIETY OF PHYSICIANS AND SURGEONS.

*Regular Meeting*, *June* 28, 1875.

(Reported by E. WARREN SAWYER, M.D.)

Dr. McKennan was elected to the chair *pro tem.*

Dr. DeWolf reported a case of rare disease of the muscular structure. The subject was a boy of fifteen years, whose previous history presented nothing unusual; he had worked in a cotton factory since the age of twelve. During the later months of 1871, he complained of a weakness in the legs and back; his mother observed that the calf of his legs were swollen. In June, 1872, he was first seen by Dr. DeWolf, when the following was noted : entire groups of muscles, as of the extensors of the foot, gastrocnemei, glutei, the erector spinæ, and those over the scapulæ, had increased in size, much beyond their usual volume. The enlargement was uniform, and the form was preserved. Photographs were exhibited, showing very noticeable enlargement of the calves. He was able to stand alone only by arching the back strongly, in order to preserve the centre of gravity. Cutaneous sensibility perfectly normal. The muscles responded to the electric current, though less readily than usual. During the fall of 1872, he was confined to the bed and was unable to turn himself; began now to complain of an ache in the anterior part of his chest. From month to month, the muscles of the upper extremity and chest were observed to become firmer, and to lose their contractility, the abdominal muscles remaining natural. The respiratory function became more and more impaired, until death, which occurred in February, 1873. The functions of the brain, digestive apparatus, heart and kidney, were normally performed to the end.

The *post mortem* examination was necessarily limited to the removal of small bits of muscle from various regions. "The apparent hypertrophy of the muscles was produced by a complete permeation of the tissue with fat, in which, however, the muscular fibre remained small and rather pale, but not affected with fatty metamorphosis." Not a simple accumulation of fat between the bundles of fibre which made up the muscle, but each muscular fibre was separated from its neighbor by a row of fat cells. "In the tissue of the shoulder, arm and forearm, a still rarer form of change had taken place. The tissue was devoid of fat, but there existed a very marked hyperplasia of the connective tissue. Thick septa, of what I conceived to be newly formed connective tissue, were interposed, not only between the primitive fibres, but between the secondary bundles of fibres, and between the muscles themselves. The muscular fibre was small, with here and there a granular appearance, but I did not observe any true fatty degeneration."

The writer then reviewed very extensively the literature and reports of analogous diseases.

In answer to Dr. Sawyer: the muscles of mastication and those of the throat were not involved. The patient died from inability to keep up the respiratory act.

The paper was further discussed by Dr. Bannister and others.

Dr. Thoms, of New York City, made some interesting remarks upon "floating hospitals," giving a history of the system, and the great benefits which have been experienced from it by hundreds of children of New York. The Dr. hopes soon to give a practical illustration of its advantages by sending a hospital boat upon Lake Michigan from our own city.

The remarks were discussed by Drs. Bartlett, Owens, DeWolf, and others.

